# First Report and Molecular Characterization of Umbra-like Virus on *Ficus carica* Caprifig Trees in Crimea

**DOI:** 10.3390/plants13162262

**Published:** 2024-08-14

**Authors:** Elena Motsar, Anna Sheveleva, Fedor Sharko, Irina Mitrofanova, Sergei Chirkov

**Affiliations:** 1 Department of Virology, Faculty of Biology, Lomonosov Moscow State University, 119234 Moscow, Russia; elena.motsar31@gmail.com (E.M.); anncsh@yandex.ru (A.S.); 2 National Research Center “Kurchatov Institute”, 123182 Moscow, Russia; fedosic@gmail.com; 3 Tsitsin Main Botanical Garden of Russian Academy of Sciences, 127276 Moscow, Russia; irimitrofanova@yandex.ru

**Keywords:** *Ficus carica* L., caprifig, fig umbra-like virus, high-throughput sequencing, plant virome

## Abstract

Fig mosaic is the most serious viral disease affecting figs. A fig germplasm collection from the Nikita Botanical Garden on the Crimean Peninsula was surveyed for viruses using high-throughput sequencing and RT-PCR with primers specific to known fig viruses. Reads related to fig umbra-like virus (FULV) were generated in samples from *Ficus carica* caprifig (pollinator) trees of the cultivar Belle dure. *F. carica* trees of other cultivars, as well as *F. afghanistanica*, *F. palmata*, and *F. virgata* trees, tested negative for FULV. Near-complete genomes of five Crimean fig umbra-like virus (FULV-CR) isolates shared 99.4% to 99.9% identity and were most closely related (85.2% identity) to the Hawaiian FULV isolate Oahu1 (MW480892). Based on their genome structure and a phylogenetic analysis, the FULV-CR isolates were determined to be dicot-infecting Class 2 umbra-like viruses and seem to be highly divergent forms of the same virus found recently in Hawaii, USA. This is the first report of an umbra-like virus found on figs in Crimea and outside of Hawaii, expanding information on the geographical distribution and genetic diversity of FULV. All of the Crimean FULV-positive plants were also co-infected with fig mosaic virus, fig badnavirus 1, and grapevine badna FI virus.

## 1. Introduction

Fig (*Ficus carica* L., family *Moraceae*) is an ancient fruit crop that is now distributed worldwide across tropical and subtropical areas [1]. Fig mosaic is the most serious disease affecting this crop. It was first reported in California [2] and is currently common in most fig-growing regions. Fig mosaic disease (FMD) can be displayed on leaves and fruits as mottling, mosaic, ring spots, discoloration, or malformation. Fruits can also be distorted and drop prematurely. The disease is spread through cuttings from diseased plants and can be transmitted by the eriophyid mite *Aceria ficus* [3,4]. 

FMD is considered to have a viral etiology; its symptoms are mainly due to fig mosaic virus (FMV, genus *Emaravirus*, family *Fimoviridae*). Fifteen viruses from different taxonomic groups have also been found in figs [5,6,7,8,9]. Since mixed virus infection is a common occurrence in fig trees, other viruses, viroids, or phytoplasmas can modulate FMV-induced manifestations of disease, generating the great symptom diversity that is typical of FMD [3]. 

Recently, fig umbra-like virus (FULV) was discovered in Hawaii, USA, on trees with FMD symptoms [10]. The complete genomes of two FULV isolates from the islands Oahu and Kauai shared 86% identity and both were classified as members of the genus *Umbravirus* in the family *Tombusviridae.*

The genus *Umbravirus* is represented by two groups of infection agents: umbraviruses [11] and umbra-like viruses (ULVs) [12]. The single-stranded positive-sense RNA genome of ULVs is 2.7 to 4.6 kb long and contains two open reading frames (ORFs) in the 5′-terminal part. ORF1 starts close to the 5′-end and encodes a replication-required protein. ORF2 encodes the RNA-dependent RNA polymerase (RdRp) and is translated as an ORF1-ORF2 fusion product that is generated by a −1 programmed frameshifting following the ORF1 translation. Frameshifting occurs due to ribosomal slippage on a conservative heptanucleotide sequence at the end of ORF1 [13,14]. ULVs encode no movement protein, but can use the host phloem protein PP2 for systemic spread through the plant [12]. 

Based on their RdRp identity, genome structure, and phylogenetic analysis, ULVs are divided into two groups. Group 1 ULVs are more related to umbraviruses. Group 2 viruses form a distinctive cluster and are subdivided into three classes. The 3′-terminal segment of Class 1 ULV genomes is a long untranslated region (UTR). Dicot-infecting Class 2 ULVs contain ORF5, which partially overlaps with the 3′-end of ORF2 and seems to encode the coat protein (CP) [12]. The genomes of monocot-infecting Class 2 ULVs have an additional ORF6 embedded in ORF5. Citrus yellow vein-associated virus belongs to Class 2 despite the lack of ORF5 [13]. Class 3 ULVs include strawberry virus A [15], grapevine umbra-like virus [16], and wheat umbra-like virus [17]. The 3′-terminal part of the genome of these viruses contains one or two ORFs that can partially overlap with ORF2 and potentially encode proteins with unknown functions [15,16,17]. Hawaiian FULV isolates (FULV-HI) are dicot-infecting Class 2 ULVs.

An old fig germplasm collection is situated in the Nikita Botanical Garden (NBG) in the subtropical area of the Crimean Peninsula. It was founded in the first half of the 19th century and now includes over 800 trees, representing 267 local and introduced *F. carica* cultivars, as well as other fig species: *F. palmata* Forssk., *F. virgata* Roxb., and *F. johannis* subsp. *afghhanistanica* (Warb.) Borowicz [18]. FMD symptoms were observed on a third of trees [19]. FMV, fig badnavirus 1 (FBV1), grapevine badna FI virus (GBFIV), and fig cryptic virus (FCV) were previously detected in the collection [7,20,21]. 

In this work, reads related to FULV were generated when studying the virome of five *F. carica* caprifig (pollinator) trees of the cultivar Belle dure using high-throughput sequencing (HTS). The near-complete genomes of five Crimean fig umbra-like virus (FULV-CR) isolates were assembled, annotated, and characterized. The Crimean and Hawaiian isolates were shown to be highly divergent forms of the same virus. This is the first report of umbra-like virus on figs in Crimea and outside of Hawaii, expanding information on the geographical distribution and genetic diversity of fig ULVs. FMV, FBV1, and GBFIV were also detected in the caprifig trees. 

## 2. Results and Discussion

**Figure 1:**
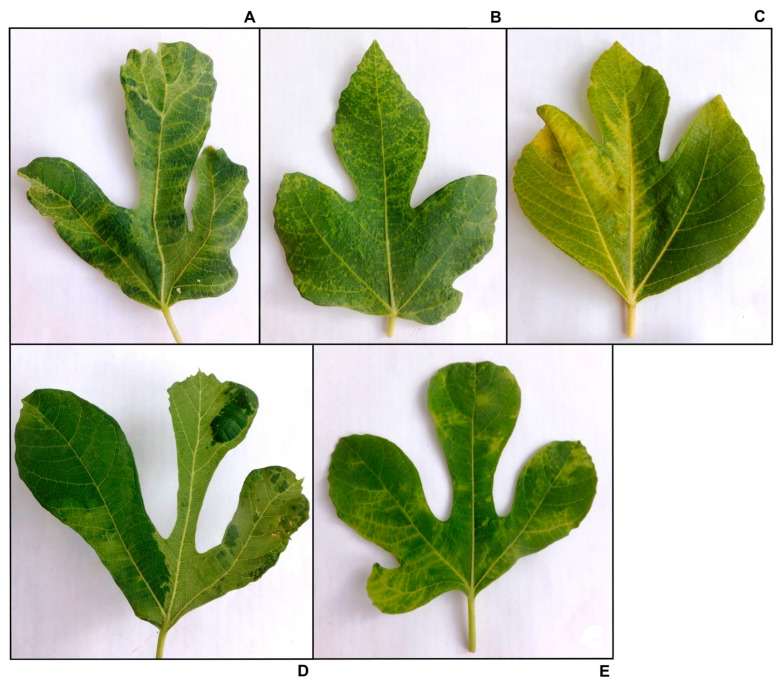
Fig mosaic disease symptoms on *Ficus carica* caprifig trees of the cultivar Belle dure. (**A**–**E**)—leaves from trees 1, 12, 23, 33, and 45, respectively.

Five caprifig trees displayed clear FMD symptoms (Figure 1). Total RNA isolated from these trees was used to conduct HTS. Reads related to FMV, FBV1, GBFIV, and FULV were generated from all plants which viromes were sequenced (Table 1). 

**Figure 2:**
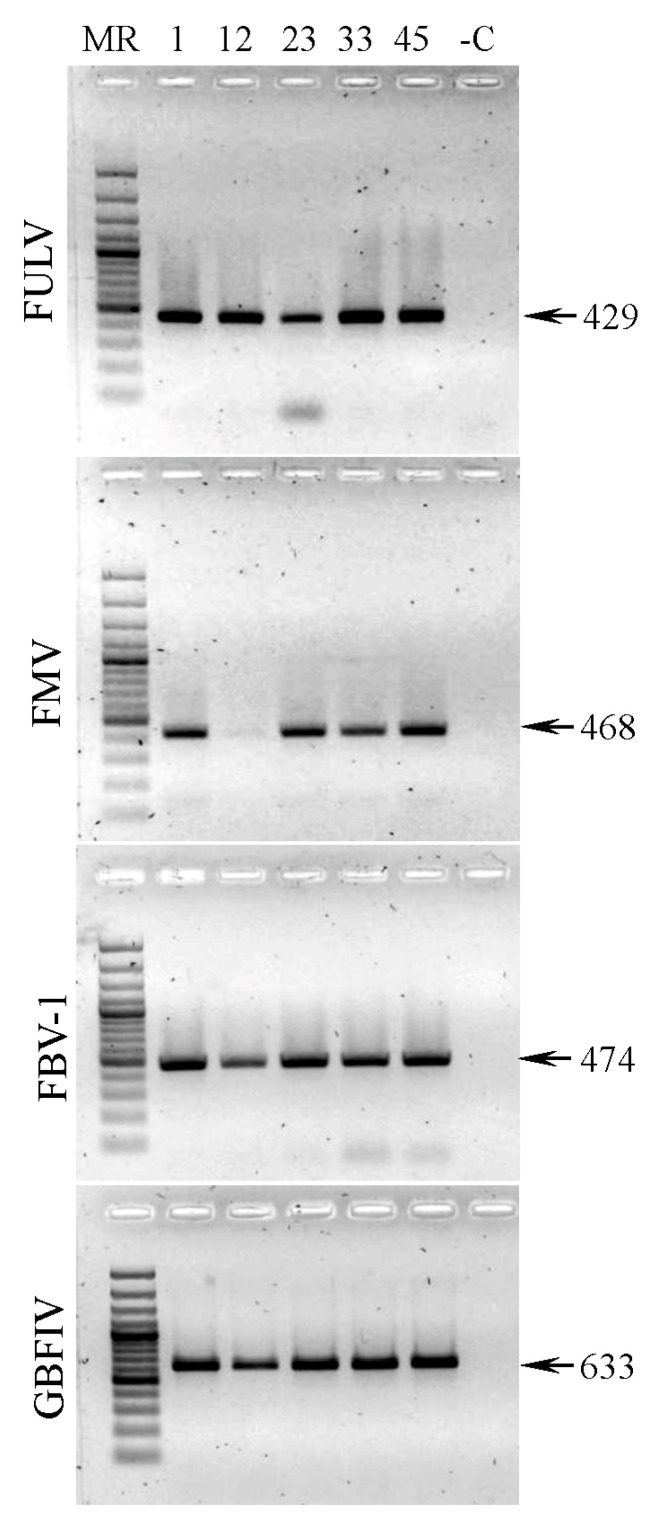
Agarose gel electrophoresis of amplicons generated by RT-PCR assay of fig umbra-like virus (FULV), fig mosaic virus (FMV), fig badnavirus 1 (FBV1), and fig badna FI virus (FBFIV) using virus-specific primers. The numbers of the trees are indicated above the picture. MR—GeneRuler 100 bp DNA ladder Plus (Thermo Scientific, Waltham, MA, USA). -C—negative control (fig tree II/2/70, infected with none of the four viruses tested [7]). Arrows to the right of the picture indicate PCR products of the corresponding size (bp).

FULV was confirmed via RT-PCR using FULV-HI-specific primers [10]. PCR products that were the expected size of 429 bp were obtained from all caprifig samples (Figure 2, top panel). Samples from *F. carica* trees of other local and introduced cultivars, as well as samples of *F. afghanistanica*, *F. palmata*, and *F. virgata* trees prepared in 2020 [7], tested negative for FULV, except for the same five caprifigs of the cultivar Belle dure. Thus, FULV was detected in caprifig samples taken in different years from different branches, suggesting that the virus was spread throughout the plants and that the infection was persistent. This is the first report of FULV in Crimea and outside of Hawaii. FMV, FBV1, and GBFIV were also verified with RT-PCR using virus-specific primers (Figure 2). 

De novo assembly yielded FULV-related contigs of 2954, 2956, 2973, 3001, and 3079 bp. BLASTn showed that they were most closely related to the complete genomes of the Hawaiian FULV isolates Oahu1 (MW480892) and Kauai1 (MW480893) (85.2% and 83.3% identity, respectively; query coverage 99%). The assembled contigs corresponded to near-complete genomes of the FULV isolates that were named BD1, BD12, BD23, BD33, and BD45 according to the tree numbers in which they were found. The identity of the BD genomes ranged from 99.4% to 99.9%. The sequences of the genome regions, determined both by HTS and the Sanger method, were identical. The near-full-length genomes of the FULV-CR isolates were deposited in GenBank under the accession numbers OR890003—OR890007. 

The BD genomes were 2953 to 3076 nt long and organized in three ORFs, which were flanked with a 5′-UTR of 9 nt and a 3′-UTR of 252 to 375 nt. Typical of ULVs, the genomes started with the carmovirus consensus sequence GGGUAAAU [14]. The ORF1 start AUG codon was in an excellent Kozak context (AAUAUGG) in all the BD isolates. ORF1 extended from 10 to 600 nt and encoded a protein of 196 amino acid (aa) residues. 

ORF2 of ULVs is known to encode the RdRp and is translated via a −1 programmed ribosomal frameshifting in close proximity to the ORF1 stop codon [23]. The potential ribosomal slippery site in the BD genomes was represented by the sequence GGAUUUC_579–585_, positioned 12 nt upstream from the ORF1-terminating UGA codon. The slippery sequence was flanked with typical upstream CA and downstream C residues. Due to ribosomal slippage, the nucleotide U_582_ was read twice, and the RdRp was translated as an ORF1-ORF2 fusion protein of 723 aa that was 5′-coterminal with the ORF1-encoded protein. The GDD motif of polymerase was found at positions 517–519 of the fusion protein. The slippery motif was confirmed using Sanger sequencing and was the same in the five FULV-CR isolates; it contained two substitutions compared to other known Class 2 ULVs (GGGUUUU). Although non-canonical, the corresponding heptanucleotide sequence in the BD genomes can presumably act as a slippery site. 

ORF5 was predicted at positions 2108 to 2701 nt. It partially overlaps the 3′-end of ORF2 and encodes a protein of 197 aa that seems to be the viral CP and is translated from subgenomic RNA [12]. The putative elements of the umbra-like Class 2 subgenomic promoter (the palindrome GAGCTC_2078–2083_ and carmovirus consensus sequence GGGUAAAA_2087–2094_) were revealed upstream from the ORF5 start AUG codon. 

**Figure 3:**
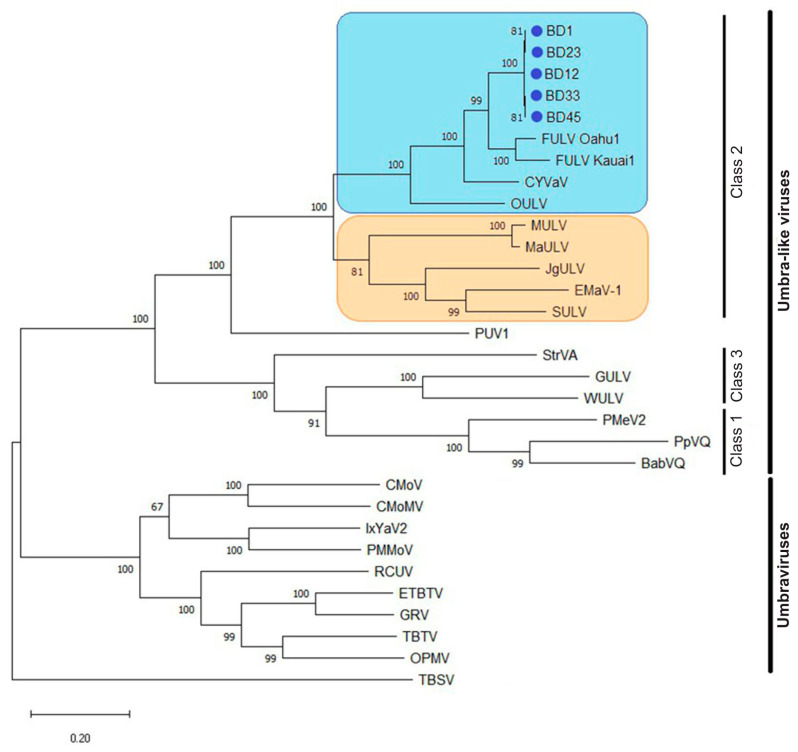
Maximum likelihood phylogenetic tree based on the complete genome sequences of umbraviruses and umbra-like viruses (ULVs) of higher plants. Bootstrap support values out of 1000 replicates (in percentage) are indicated next to the corresponding nodes. The virus acronyms are shown at the end of the branches. Two clusters (ULVs and umbraviruses) and three classes of ULVs (1, 2, and 3) are highlighted. ULVs from dicot and monocot hosts are shaded blue and orange, respectively. The scale bar indicates nucleotide substitutions per site. Tomato bushy stunt virus (TBSV, MW582792) was used as a phylogenetic outgroup. The abbreviated names and accession numbers of the viruses are as follows: ULVs (top to bottom): BD1, BD12, BD23, BD33, and BD45, highlighted by a blue circle (●)—Crimean fig umbra-like (FULV) isolates (OR890007, OR890003, OR890004, OR890005, OR890006, respectively); FULV Oahu1 and FULV Kauai1—Hawaiian FULV isolates Oahu1 (MW480892) and Kauai1 (MW480893); CYVaV—citrus yellow-vein-associated virus (JX101610); OULV—opuntia umbra-like virus (MH579715); MULV—maize umbra-like virus (OM937759); MaULV—maize-associated umbra-like virus (OK018180); JgULV—johnsongrass umbra-like virus (OM937760); EMaV-1—Ethiopian maize-associated virus (MN715238); SULV—sugarcane umbra-like virus (MN868593); PUV1—parsley umbravirus 1 (OM419177); StrVA—strawberry virus A (MK211274); GULV—grapevine umbra-like virus (OP886321); WULV—wheat umbra-like virus (OK573479); PMeV2—papaya meleira virus 2 (KT921785); PpVQ—papaya virus Q(KP165407); BabVQ—babaco virus Q (MN648673). Umbraviruses (top to bottom): CMoV—carrot mottle virus (FJ188473); CMoMV—carrot mottle mimic virus (U57305); IxYaV2—ixeridium yellow mottle-associated virus 2 (KT946712); PMMoV—Patrinia mild mottle virus (MH922775); RCUV—red clover umbravirus (MG596237); ETBTV—Ethiopian tobacco bushy top virus (KJ918748); GRV—groundnut rosette virus (MG646923); TBTV—tobacco bushy top virus (KX216407); OPMV—opium poppy mosaic virus (EU151723).

A phylogenetic analysis of the BD isolates was performed using the full-length genome sequences of all available umbraviruses and ULVs from higher plants retrieved from GenBank (Figure 3). Umbraviruses and ULVs formed two distinct well-supported clusters. ULVs were grouped into three clades corresponding to Class 1, 2, and 3 ULVs. BD isolates were assigned to a separate clade within the Class 2 group. The sister clade consisted of the FULV-HI isolates, indicating a common origin of the fig-infecting ULVs. Based on the genome structure and phylogenetic analysis, it was determined that FULV-CR is a dicot-infecting Class 2 ULV, similar to the Hawaiian isolates.

The complete FULV-CR genomes were 83–85% identical to the FULV-HI isolates. The species demarcation criterion in the genus *Umbravirus* is a nucleotide sequence identity of less than 70% [24]. The Hawaiian isolates Oahu1 and Kauai1 were 86% identical to each other and considered the same virus [10]. The BD isolates were successfully detected using RT-PCR with FULV-HI-specific primers. Collectively, the Crimean isolates seem to be a divergent FULV form rather than a new virus species. 

The coding genome regions and 3′-UTRs of the Crimean isolate BD1 and the Hawaiian isolates Oahu1 and Kauai1 were compared (Table 2). The BD1 isolate was only used for comparison purposes as the Crimean isolates were practically identical to one another. Depending on the ORFs, the Crimean and Hawaiian isolates shared 79.3% to 90.3% and 76.3 to 93.5% identity at the nt and aa levels, respectively. The differences were mainly due to mutations randomly dispersed along the genome. In ORF1 and ORF5, variability was more pronounced at the aa level, suggesting that many mutations were non-synonymous. On the contrary, the RdRp was more conservative at the aa level. The ORF1 and ORF2 of BD1 were 6 nt and 2 aa longer than those of Oahu1 and Kauai1. Two mismatches were found in the slippery motifs of BD1 (GGAUUUC) compared to the Oahu1 and Kauai1 isolates (GGGUUUU). Also, the sequences of the putative subgenomic promoter elements in the Oahu1, Kauai1, and BD1 isolates were slightly different. The 6 nt long palindrome was represented by the sequences GAGCTC and GGGCCC in the Crimean and Hawaiian isolates, respectively. The 3′-UTRs diverged most significantly.

The molecular diversity of the Oahu1 and Kauai1 isolates was proposed to result from the different geographic sample origins or due to corresponding to different strains of the same viral species [10]. It is possible that the BD isolates represent another geographically distant FULV strain inhabiting climatic conditions similar to the Hawaiian isolates. 

The caprifig of the cultivar Belle dure was imported to the NBG from France in 1918 [25] and has periodically been renewed through propagation by own-root cuttings since then. Caprifigs are pollinator plants that are required to increase pollination efficacy [26]. The FULV-CR isolates were detected only on caprifigs and not in the other *F. carica* cultivars or fig species. It is highly likely that the imported caprifig was infected with FULV, suggesting that this virus can also occur in fig-growing regions other than Hawaii and Crimea. This could also explain the low genetic diversity of the FULV-CR isolates, which appear to be the progeny of a single isolate introduced into the NBG via an infected cutting. Why the virus spread was limited to only one fig cultivar remains to be determined. Umbraviruses encode no CP and therefore need a helper luteovirus/enamovirus/polerovirus/sobemovirus to achieve vector-mediated transmission to another plant [11,24]. No HTS-generated reads that could be attributed to a helper virus were revealed in the Crimean caprifig samples. No helper virus was found in the Hawaiian FULV-HI-infected trees, either [10]. On the other hand, the ORF5 of the Class 2 ULVs seems to encode the CP [12] and Class 3 wheat umbra-like virus was shown to be transmissible by sap inoculation [17]. These findings suggest that the transmission of ULVs from plant to plant may not rely on a helper virus and highlight the need for a large-scale survey of the prevalence of FULV in the NBG’s collection. The spread of the virus could also be precluded by the lack of a specialized vector that has yet to be identified.

Apart from FULV, the caprifigs were co-infected with other viruses. Reads related to FMV, FBV1, and GBFIV were generated using HTS. Complete FMV and FBV1 genomes were assembled in the samples from the trees 1, 12, 23, and 33. Typical of the genus *Emaravirus*, FMV genomes were represented by six single-stranded RNAs. Depending on the RNA segment, the caprifig isolates were 98.4% to 99.9% identical to each other and 99.2% to 99.7% identical to the FMV isolates from the NBG that were characterized previously [20]. At the same time, the identity of the Crimean FMV isolates with those from other countries was noticeably lower irrespective of the genome segment, suggesting that the caprifigs could have been infected with FMV isolates from neighboring fig trees. The full-length FMV genome sequences were deposited in GenBank under the accession numbers PP196567–PP196575, PP197702–PP197713, and PP228020–PP228022. The Crimean full-length FBV1 genomes were assembled for the first time. They were 99.9–100% identical to one another, in agreement with a previous report indicating the extremely low genetic diversity of FBV1 isolates from different localities [27], and were deposited in GenBank under the accession numbers PP196563–PP196566. Multiple GBFIV-related contigs ranging from 250 to 1275 nt were obtained from all five samples. They were 99.3–100% identical to the corresponding genome regions of the most closely related GBFIV isolate Blu17 (OP087316) from the NBG’s fig collection [7]. 

Both Crimean and Hawaiian FULV isolates were found in the symptomatic trees. However, the contribution of FULV-CR to FMD symptoms could not be established due to the co-infection of caprifigs with different viruses, including FMV. It was also not reported whether other viruses have been found in FULV-infected trees in Hawaii [10]. 

## 3. Materials and Methods

Leaves displaying FMD symptoms were gathered from the NBG’s fig germplasm collection (N44.510941; E34.232675) in June 2022. The samples were taken from five 30–32-year-old *F. carica* caprifig trees of the cultivar Belle dure numbered 1, 12, 23, 33, and 45 according to their position on the plot. Individual samples comprising three to five leaves were collected from randomly selected branches of each tree. The samples were bagged, labeled, and delivered to the Virology Department of Lomonosov Moscow State University, where they were stored at 4 °C until further processing. Total RNA was extracted from fresh leaves using a CTAB-based method [28] and kept at −70 °C until use. 

Five cDNA libraries were synthesized using the TruSeq Stranded Total RNA Library Prep Plant kit (Illumina, San Diego, CA, USA) and caprifig RNA as the template. The libraries were sequenced on the Illumina MiSeq platform. The raw pair-ended reads of 150 bp were deposited in NCBI Sequence Read Archive (https://www.ncbi.nlm.nih.gov/sra/PRJNA1040480, accessed on 15 November 2023). To remove adapters and filter by quality, the reads were processed using FastQC v.0.11.9 and Trim Galore v.0.6.5 (https://www.bioinformatics.babraham.ac.uk/projects/trim_galore, accessed on 1 August 2023) with default parameters. The fast annotation of the sequenced reads was achieved using the Kraken 2 program [22]. Contigs were assembled de novo using the metaSPAdes program version 3.15 [29]. Virus-related contigs were identified with NCBI BLASTn (https://blast.ncbi.nlm.nih.gov/Blast.cgi, accessed on 10 August 2023) against the GenBank nucleotide collection. 

Total RNA from fig leaves was also used in RT-PCR assays of the viruses to confirm the HTS results. Random hexamer primers and MMLV reverse transcriptase (Evrogen, Moscow, Russia) were used for the first-strand cDNA synthesis. The proofreading Encyclo DNA polymerase (Evrogen) was used for PCR. Amplicons were analyzed using 1.5% (*w*/*v*) agarose gel electrophoresis, visualized using ethidium bromide staining, and photographed using the gel documentation system MultiDoc-It (Analytik Jena US LLC, Upland, CA, USA). PCR products were purified from agarose gel using BC022 Cleanup Standard kit (Evrogen) and sequenced bidirectionally using Evrogen facilities. 

To verify the presence of FULV, FMV, FBV 1, and FBFIV in the caprifig trees using RT-PCR, primers specific to the corresponding viruses were employed according to the original protocols [7,10,27,30]. In addition, thirty-one *F. carica* trees of other local and introduced cultivars, as well as one *F. afghanistanica*, five *F. palmata*, and ten *F. virgata* trees, were tested for FULV; the total RNA was prepared in 2020 [7] and kept at −70 °C. To validate the FULV genome sequences determined with HTS, several primer pairs, based on the near-complete genome of the FULV-CR isolates, were designed (Table S1) and used for PCR and Sanger sequencing. 

To analyze the FULV-CR genomes, all available full-length genome sequences of umbraviruses and plant ULVs were retrieved from GenBank. Multiple alignments of nt sequences using ClustalW v. 1.81, sequence identity calculation, and phylogenetic analysis were performed in MEGA X v. 10.2.5 [31]. The phylogenetic tree was reconstructed using the maximum likelihood method and the Tamura–Nei model. Bootstrap values were calculated using 1,000 pseudo replications. The ORFs were identified using the NCBI ORF finder (https://ncbi.nlm.nih.gov/orffinder, accessed on 3 June 2024). Conserved domains in virus proteins were mapped using the Conserved Domain Database (CDD, https://ncbi.nlm.nih.gov/Structure/cdd/wrpsb.cgi, accessed on 3 June 2024) algorithm. 

## 4. Conclusions 

Sequences resembling umbraviruses were previously found in figs from California using Sanger sequencing for dsRNAs and RT-PCR with primers specific to the RdRp of carrot mottle mimic umbravirus [30]. The first fig umbra-like virus with a fully characterized genome was FULV, originating from Hawaii [10]. Our work is the first report of an umbra-like virus on figs in Crimea and outside of Hawaii, thus expanding the information on the geographical distribution and genetic diversity of fig ULVs. The Crimean and two Hawaiian FULV isolates may be different strains of the virus. 

## Figures and Tables

**Table 1:** Characterization of reads ^a^ generated from the caprifig trees by high-throughput sequencing.

Tree Number	Total Reads	Viral Reads ^b^, %	Reads ^b^ Related to			
			FMV	FBV1	FULV	GBFIV
1	11,140,831	3.111	344,864	339	151	75
12	7,559,227	0.916	68,366	210	361	44
23	11,682,485	1.574	182,556	485	88	73
33	12,373,421	3.425	422,004	359	328	71
45	12,762,621	0.552	69,792	237	454	78

^a^ 2 × 150 bp; ^b^ calculated using Kraken 2 program [22]. FMV—fig mosaic virus; FBV1—fig badnavirus 1; FULV—fig umbra-like virus; GBV1—grapevine badnavirus 1.

**Table 2:** Identity of open reading frames (ORFs) (nt/aa, %) and 3′-untranslated region (3′-UTR) (nt, %) of the Crimean and Hawaiian fig umbra-like virus isolates BD1, Oahu1, and Kauai 1.

Virus Isolate	Oahu1				Kauai1			
	OFR1	ORF2	ORF5	3′-UTR	OFR1	ORF2	ORF5	3′-UTR
BD1	80.0/76.8	85.5/87.8	85.7/83.8	76.6	79.3/76.3	83.7/86.3	83.2/81.7	55.3
Oahu1	x				89.7/90.7	90.3/93.5	88.9/90.4	47.5

## Data Availability

The sequencing data were deposited in GenBank and the NCBI Sequence Read Archive. Their accession numbers are provided within the article.

## Supplementary Materials

The following supporting information can be downloaded at https://www.mdpi.com/article/10.3390/plants13162262/s1: Table S1: Primers for the Sanger sequencing of Russian FULV isolates.
