# Supplementary material for: First Report and Molecular Characterization of Umbra-like Virus on Ficus carica Caprifig Trees in Crimea
Source: Plants (Basel). 2024 Aug 14;13(16):2262. doi: 10.3390/plants13162262 (PMC11359341; doi:10.3390/plants13162262)
Supplement: Supplementary file 1 [file plants-13-02262-s001.zip › plants-3086954-supplementary.pdf]

Supplementary Material

First Report and Molecular Characterization of Umbra-like Virus on *Ficus carica* Caprifig Trees in Crimea

Elena Motsar [1], Anna Sheveleva [1], Fedor Sharko [2], Irina Mitrofanova [3] and Sergei Chirkov [1,*]

* Correspondence: s-chirkov1@yandex.ru

Table S1. Primers for the Sanger sequencing of Russian FULV isolates.

Primer name	Primer sequence, 5'..3'	Genome positions	PCR product, bp	Reference
5'UTR-fulv-F	AAGGGTAAAATATGGAAACAGT	1 - 20	418	This work
5'UTR-fulv-R	TCTACACCGTTGAGTTGTTTGA	397 - 419		
UL1-F	GTTGGATGGGCACTTCGTCT	313 - 332	1148	This work
Fig-UmbraR	TGTCCATGTCGCCACTCATT	1442 - 1461		
Fig-UmbraF*	TGAAGGGAGACGAGGCTGAT	1042 - 1061	429	[10]
Fig-UmbraR*	TGTCCATGTCGCCACTCATT	1442 - 1461		
fulv-F1	CAAGTCTCCCACTCTTCAAAGA	1349 - 1370	636	This work
fulv-R1	CTCAACTTCAGGTTGTCTGTCCAT	1962 - 1985		
fulv-F2	ATTGGGTACGAATTCGCGTATAGA	1893 - 1915	623	This work
fulv-R2	AGCGCCGCGTATAGTTACAAGA	2495 - 2516		
fulv-F3	TGGGGTTGGTCTCAGTACTTCT	2413 - 2434	338	This work
fulv-R4	AGTCCGGTAGGAGAGCAGTA	2732 - 2751		

*Primers were used for FULV detection by RT-PCR
